# A method of large DNA fragment enrichment for nanopore sequencing in region 22q11.2

**DOI:** 10.3389/fgene.2022.959883

**Published:** 2022-10-31

**Authors:** Yu-Qing Lei, Liang-Pu Xu, Hua Cao, Xin-Rui Wang

**Affiliations:** ^1^ Fujian Maternity and Child Health Hospital, College of Clinical Medicine for Obstetrics and Gynecology and Pediatrics, Fujian Medical University, Fuzhou, China; ^2^ NHC Key Laboratory of Technical Evaluation of Fertility Regulation for Non-human Primate (Fujian Maternity and Child Health Hospital), Fuzhou, China; ^3^ Department of Cardiac Surgery, Fujian Children’s Hospital (Fujian Branch of Shanghai Children’s Medical Center), College of Clinical Medicine for Obstetrics and Gynecology and Pediatrics, Fujian Medical University, Fuzhou, China; ^4^ Medical Genetic Diagnosis and Therapy Center, Fujian Maternity and Child Health Hospital, College of Clinical Medicine for Obstetrics and Gynecology and Pediatrics, Fujian Medical University, Fuzhou, China; ^5^ Medical Research Center, Fujian Maternity and Child Health Hospital, College of Clinical Medicine for Obstetrics and Gynecology and Pediatrics, Fujian Medical University, Fuzhou, China

**Keywords:** region-specific amplification, nanopore sequencing, 22q11.2 deletion syndrome, genomic analyses, target region enrichment method

## Abstract

**Background:** 22q11.2 deletion syndrome (22q11.2DS) is a disorder caused when a small part of chromosome 22 is missing. Diagnosis is currently established by the identification of a heterozygous deletion at chromosome 22q11.2 through chromosomal microarray analysis or other genomic analyses. However, more accurate identification of the breakpoint contributes to a clearer understanding of the 22q11.2 deletion syndrome.

**Methods:** In this study, we present a feasible nanopore sequencing method of 22q11.2 deletion. This DNA enrichment method—region-specific amplification (RSA)—is able to analyze the 22q11.2 deletion by specific amplification of an approximately 1-Mb region where the breakpoint might exist. RSA introduces universal primers into the target region DNA by a Y-shaped adaptor ligation and a single primer extension. The enriched products, completed by amplification with universal primers, are then processed by standard ONT ligation sequencing protocols.

**Results:** RSA is able to deliver adequate coverage (>98%) and comparable long reads (average length >1 Kb) throughout the 22q11.2 region. The long nanopore sequencing reads, derived from three umbilical cord blood samples, have facilitated the identification of the breakpoint of the 22q11.2 deletion, as well as by Sanger sequencing.

**Conclusion:** The Oxford Nanopore MinION sequencer can use RSA to sequence the target region 22q11.2; this method could also be used for other hard-to-sequence parts of the genome.

## Introduction

22q11.2 deletion syndrome (22q11.2DS) is a disorder caused when a small part of chromosome 22 is missing. Medical problems associated with 22q11.2 deletion syndrome commonly include heart defects, poor immune system function, a cleft palate, complications related to low levels of calcium in the blood, and delayed development with behavioral and emotional problems (McDonald-McGinn et al., 1999; McDonald-McGinn et al., 2015). The diagnosis of 22q11.2DS is established by the identification of a heterozygous deletion at chromosome 22q11.2 using chromosomal microarray analysis or other genomic analyses (McDonald-McGinn et al., 1999; McDonald-McGinn et al., 2015). None of these methods can accurately assess the breakpoint, thereby making them also unable to accurately assess the involved genes.

The Oxford Nanopore MinION sequencer, known as a “third-generation” sequencing platform, is capable of producing significantly longer read lengths and analyzing traditionally problematic sequence regions with high GC content (Mikheyev and Tin, 2014). The longer read lengths have been shown to be highly advantageous in identifying structural variants and haplotype phasing within complex genomic loci (Ritz et al., 2014; Wang et al., 2015).

Targeted sequencing enables researchers to enrich loci of interest, thereby reducing sequencing costs and labor, and facilitating high coverage data for genomic regions of interest. For next-generation sequencing, the most common strategy is amplification or hybridization capture (Kozarewa et al., 2015). However, none of these take advantage of new long-read sequencing technologies such as nanopore because long-fragment amplicons with relatively large bias and hybridization capture have yet to be fully optimized for long fragments. Long-fragment PCR and CATCH-seq3, in which regions of interest are excised by dual Cas9 cleavage and then enriched by size selection, have been described for target enrichment with nanopore sequencing (Gabrieli et al., 2018; Gilpatrick et al., 2020); however, these methods are not suitable for the enrichment of larger regions (>1 Mb).

**FIGURE 1:**
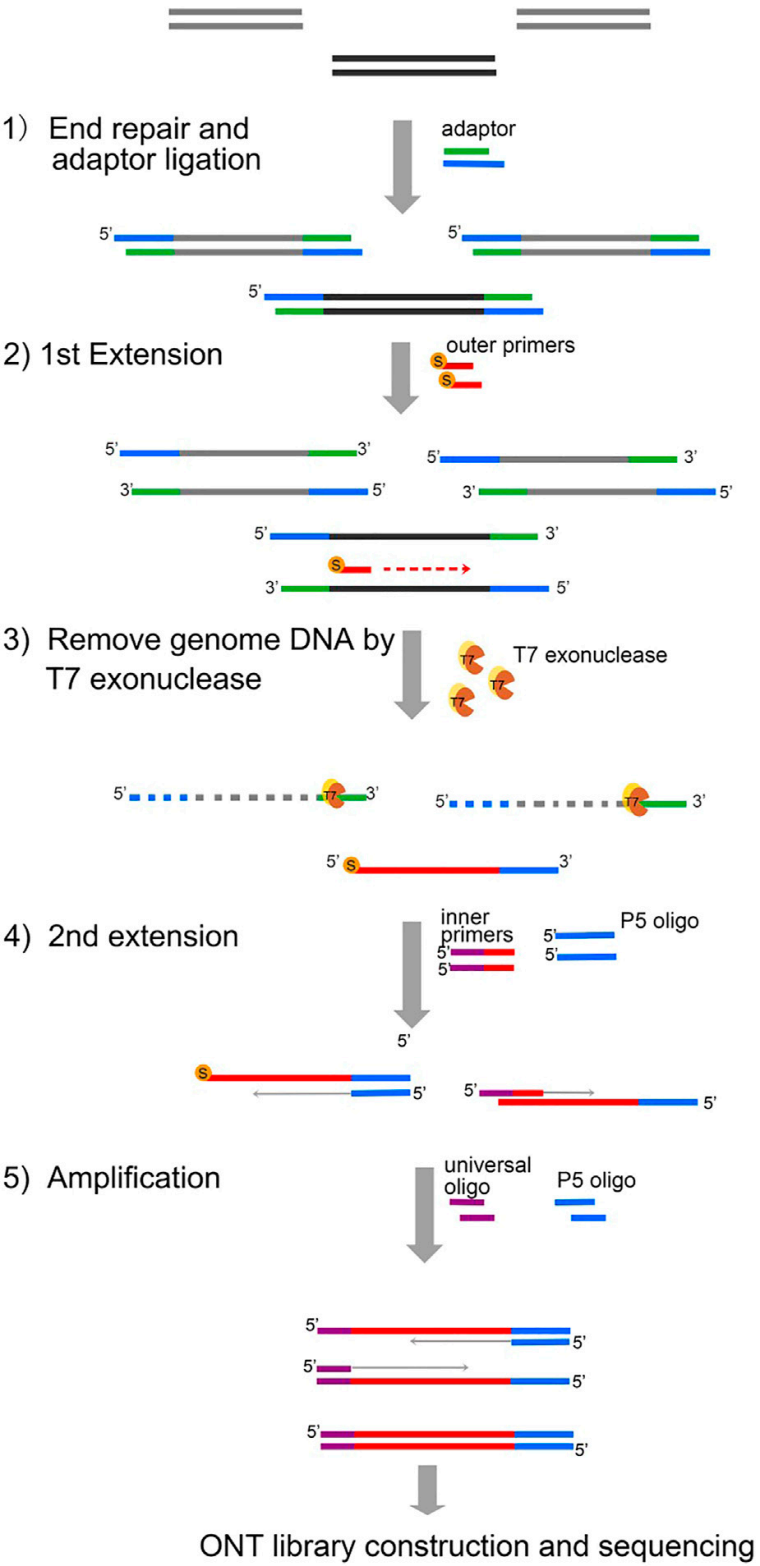
Principle of RSA. 1) During the first step of RSA, the genomic template DNA (gray) performed end repair and adaptor ligation using a DNA Library Kit for Illumina. 2) First extension by the outer primers modified with 5′ thiophosphorylation. 3) Genomic DNA was removed by T7 exonuclease. The newly generated product cannot be degraded from T7 due to protection by the 5′ thiophosphorylation modification. 4) Second extension. The second strand was generated with the P5 oligo. The second extension was completed with inner primers which are composed of universal primer sequence (purple) and specific sequences (red). 5) Amplification by the p5 oligo and universal oligo. S, thiophosphorylation. T7, T7 exonuclease.

Our DNA enrichment method, region-specific amplification (RSA), addresses this unmet need by semi-nested long DNA fragment amplification of 1–1.5 Kbp in length. The first step of RSA is adaptor ligation by the NGS library construction kit. The targeted genomic DNA segments are then extended by 5′-end thiophosphorylated primers. The majority of the genomic DNA was cleared using the T7 exonuclease. To improve the specificity, the targeted genomic DNA segments are then extended by the inner primers, with the universal sequence at the 5′-end being extended for a second time. Thus, the DNA fragments of the target region are successfully connected with a universal sequence. After limited-step amplification using universal primers, the enriched products are then processed by standard ONT ligation sequencing protocols (Figure 1).

Here, we demonstrate the utility of RSA for the targeted sequencing of the region 22q11.2 by the Oxford Nanopore MinION sequencer, which may also be applied to other complex regions of the genome.

## Methods

### Sample source

The samples used in this study were collected at Fujian Maternity and Child Health Hospital between January and December 2019. The cord blood cells were collected from patients with a gestational age of 24–35 weeks. The 22q11 microdeletion was confirmed by the Affymetrix CytoScan 750K Array with umbilical cord blood cells from pregnant women who had been diagnosed with cardiac malformation by routine prenatal ultrasound. The SNP-array results were performed by the pathology department. Written informed consent for the study using these samples was obtained from the patients and approved by the Ethics Committee of Fujian Maternal and Child Health Hospital.

### Genomic DNA preparation from umbilical cord blood cells

DNA extraction was performed with the TIANamp Blood DNA Kit (TIANGEN, Cat. no. 4992207) according to the manufacturer’s protocol, using a 200-μL umbilical cord blood sample for each reaction.

### RSA primer design

The primer design software Primer3 (Untergasser et al., 2012) was applied to design the primers, which were spaced 1–1.5 Kbp apart. When they were settled, the homology between the selected primers and the rest of the genome was checked with BLAT (Kent, 2002). In this experiment, 819 primers were designed to target the entire 1 Mbp of the 22q11 at an average spacing of ≈1 Kbp. The first two bases of all the first extension primers (outer primers) were modified by thiophosphorylation. All the second extension primers (inner primers) had a common sequence at the 5′-end (see Additional File 1: Supplementary Table S1 for the list of primers and supporting information). The primers were synthesized by Sangon Biotech (Shanghai) and were then combined (in water) to an equimolar ratio of all primers.

### Region-specific amplification

#### End repair and adaptor ligation

End repair and adaptor ligation were performed using the QuarPrep Ultra DNA Library Kit for Illumina (Dynasty gene, Cat. no. L1001A) using 200 ng genomic DNA. This step was modified from the manufacturer’s protocol: the input DNA was not fragmented, and the final amplification step was skipped.

#### Primer extension and genomic DNA removal

For each 20 μL annealing reaction, the ligation products were combined with 5 μM first extension primer mixture (outer primers), 1× NEB CutSmart Buffer, 0.4 mM dNTPs, and DNase-free water. The mixed annealing reaction was performed in a sterile DNase-free microfuge tube. The DNA/primer was denatured at 95°C for 5 min, briefly spined, and quickly placed on ice. After adding eight units of Bst 2.0 WarmStart DNA polymerase (NEB, Cat. no. M0538), the samples were incubated at 60°C for 10 min and 80°C for 20 min. Ten units of T7 exonuclease (NEB, Cat. no. M0263) were then added and incubated at 25°C for 30 min. The targeted DNA was purified by 1× AMPure XP beads.

#### Semi-nested extension

For each 20 μL annealing reaction, the ligation products were combined with 5 μM second extension primer mixture (inner primers), 5 μM P5 oligo, 1× NEB CutSmart Buffer, 0.4 mM dNTPs, and DNase-free water. The mixed annealing reaction was performed in a sterile DNase-free microfuge tube. The DNA/primer was denatured at 95°C for 5 min, briefly spined, and quickly placed on ice. After adding eight units of Bst 2.0 WarmStart DNA polymerase (NEB, Cat. no. M0538), the samples were incubated at 60°C for 20 min and at 80°C for 20 min. The targeted DNA was purified by using 1× AMPure XP beads.

#### Amplification

The primer extension products were amplified with the introduced sequences. For each 25 μL amplification reaction, the primer extension products were combined with 1× KAPA HiFi HotStart ReadyMix (Roche Sequencing Store, Cat. no KK2602), 0.4 μM each of P5 oligo, and universal oligo. The thermocycling protocol was 10 min at 94°C for the initial denaturation, followed by 25 cycles of 30 s at 94°C, 30 s at 60°C, 5 min at 72°C, and a final extension for 5 min at 72°C. The amplification products were purified using 1× AMPure XP beads.

### ONT library construction and sequencing

The ONT library was constructed according to the PCR Barcoding Kit protocol (Oxford Nanopore, Cat. no. SQK-PBK004) using 200 ng amplification products. Sequencing was performed using a R9.4 flow cell on a MinION device (Oxford Nanopore) with the MinKNOW.

### Sanger sequencing

To validate the variant calls, genomic DNA was applied as a template. PCR primers for each variant were designed manually *via* the primer-design software Primer3. The full list of PCR primers is provided in Additional File 2: Supplementary Table S2. To set up each PCR reaction, 50 ng of gDNA DNA was combined with 1×KAPA HiFi HotStart ReadyMix (Roche Sequencing Store, Cat. no KK2602), 0.4 μM each of forward and reverse primers, and water. The thermocycling protocol is 10 min at 94°C for the initial denaturation, followed by 35 cycles of 30 s at 94°C, 30 s at 55°C, 60 s at 72°C, and a final extension for 5 min at 72°C. The amplification was validated by 1% agarose gel electrophoresis. Each amplicon was then purified with the QIAquick Gel Extraction Kit (Qiagen, Cat. no. 28704). Sanger sequencing was performed at Sangon Biotech (Shanghai) using the same primers for PCR amplification.

### Sequence alignment and variant detection

Sequencing data were generated by a R9.4 flow cell on a MinION device (Oxford Nanopore) with MinKNOW. Basecalling was performed with Guppy (v.4.2.2). The reads were trimmed using Porechop (https://github.com/rrwick/Porechop) and were then aligned to the reference genome (Hg19) using Minimap2 (Li, 2018) with parameters "-a -x map-ont --MD.” After alignment, structure variant calling was fulfilled using Sniffles (Sedlazeck et al., 2018) (v1.0.12, https://github.com/fritzsedlazeck/Sniffles) with parameters "--max_distance 4000000 --minmapping_qual 10."

## Results

### Statistics for sequence data

**FIGURE 2:**
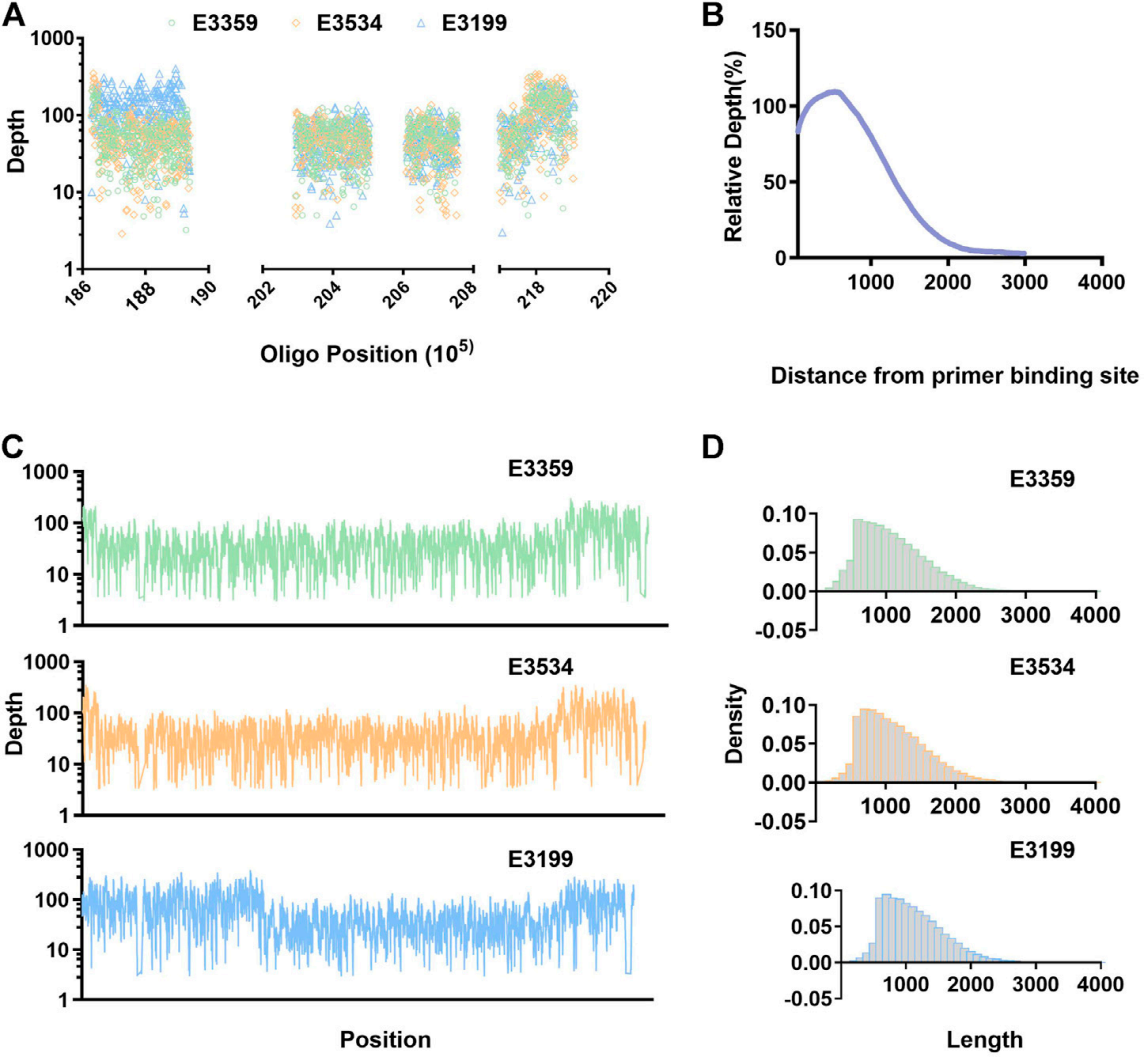
Distribution of reads. **(A)** Depth of coverage at the site of primers. **(B)** Schematic representation of the distribution of relative depth obtained after the primer hybridization site. **(C)** Sequencing coverage is displayed for the entire enriched about 1 Mb of the selected part 22q11.2 region. **(D)** Distribution of reads length.

Raw data (fastq) files have been made publicly available and are accessible through the NCBI SRA website (SRA accession: SRP326421). In all three samples, more than 30% of the reads are mapped in the target region, ensuring that sufficient valid data could be generated. A majority of the targeted region maintains high coverage depth, and the coverage rate of 5× or above is more than 95%. The average read length is more than 1 kB, and longer reads are presented—beneficial for detecting SV variation (Table 1; Figure 2D).

**TABLE 1:** Sequencing results.

	E3359	E3534	E3199
Reads	47,966	48,019	64,270
Reads (>Q10)	27,295(56.9%)	26,153(54.5%)	35,789(55.7%)
Total bases	55,341,275	57,602,066	76,462,240
Coverage rate in targeted region (depth>1)	98.3%	98.4%	98.5%
Coverage rate in targeted region (depth>5)	96.4%	95.7%	96.8%
Mapped reads to whole genome (%)	98.6%	98.7%	98.8%
Mapped reads to targeted region (%)	43.2%	43.7%	31.4%
Average depth in targeted region	51.4	53.5	50.5
Average length pg region	1154	1,200	1,190

### Distribution of reads

Most of the primer binding regions have a high depth, indicating that the primers hybridized to a specific region during annealing, and extension then occurred as expected (Figure 2B). Statistical analysis was conducted on the relative depth of the relative primer positions; it was found that the distribution was basically semi-normal. The relative depth decreased further away from the primer hybridization site. Interestingly, we found that the relative depth was highest at about 400 bp from the primer binding site, rather than at the nearest location (Figure 2A). This may be due to short DNA molecules having been filtered in the experiment and part of the amplified product being degraded or broken at the ends. The average length of reads was more than 1 kB, so we chose primer spacing of 1–1.5 Kb to achieve high coverage of the target region. This was confirmed by the coverage and depth analysis (Figure 2C, Supplementary Table S3), although this may not be the best spacing.

### Variant analysis and Sanger validation

**FIGURE 3:**
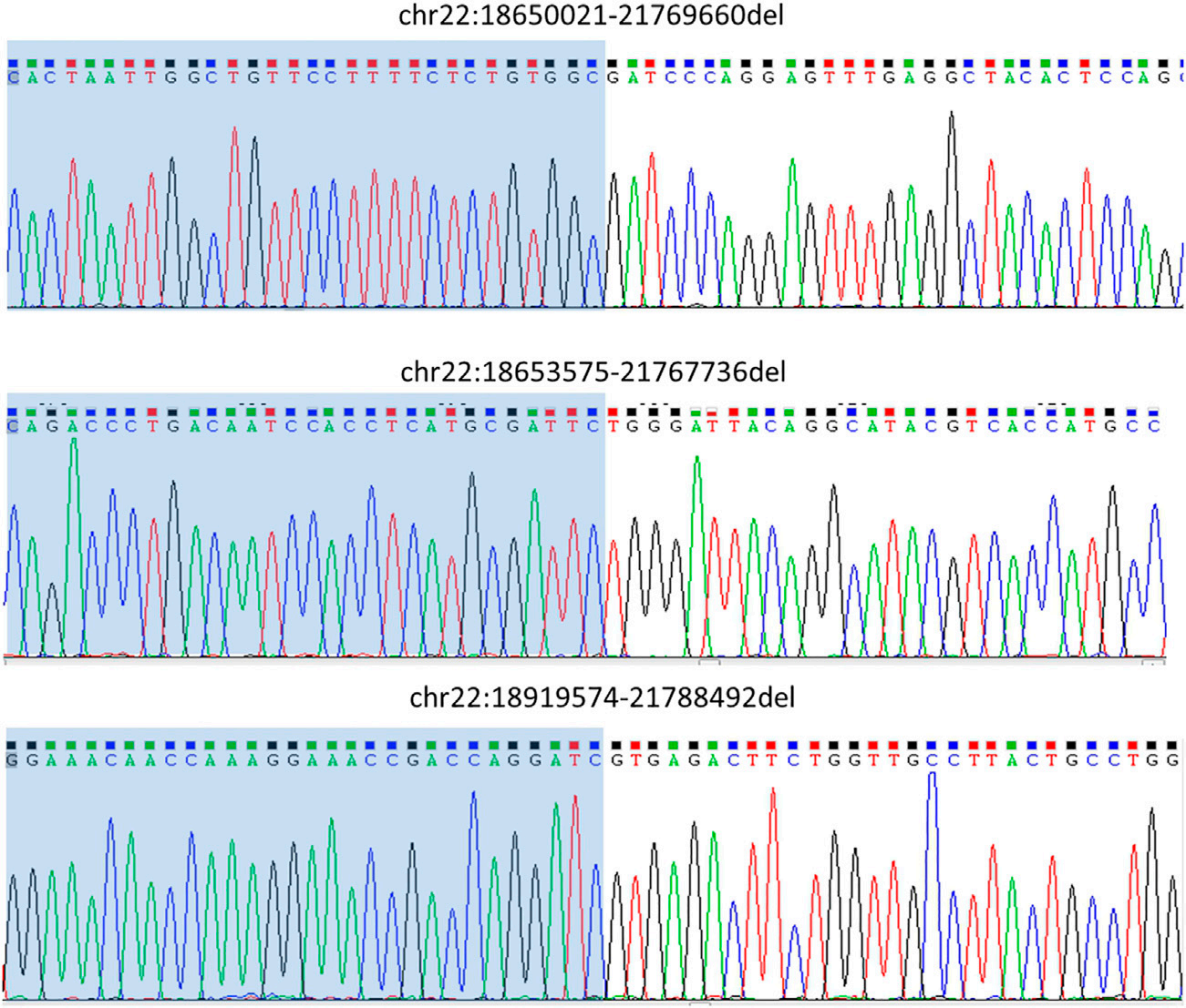
Variant validation by Sanger sequencing. Primers that were designed at both ends of the breakpoints were to perform PCR and Sanger sequencing. The Sanger sequencing results supported the Sniffles analysis results.

The sequencing results were then analyzed using Sniffles to detect structural variations. We got breakpoints for all three samples. The results were consistent with the SNP-array results. We then designed primers (Table 2) at both ends of the breakpoints to perform PCR and Sanger sequencing. The Sanger sequencing results supported the analysis and Sniffles analysis results (Figure 3, Supplementary Figure S1–S3, Supplementary Table S4). This indicates that the RSA can effectively enrich the structural variation areas.

**TABLE 2:** Results of structural variation analysis.

Sample	ONT sequencing	SNP array
E3359	22:18650021-21769660del	arr[hg19]
		22q11.21(18,648,855–21,800,471)x1
E3534	22:18653575-21767736del	arr[hg19]
		22q11.21(18,648,855–21,800,471)x1
E3199	22:18919574-21788492del	arr[hg19]
		22q11.21(18,916,842–21,800,741)x1

## Discussion

Current target-region enrichment methods are mostly based on DNA hybridization to capture an oligonucleotide (Gnirke et al., 2009) or multiplexed PCR amplification reaction (Tewhey et al., 2009). These methods, which were previously applied in NGS, can only produce relatively short (less than 1,000 base pair) sequencing templates. As a result, they cannot complete the sequencing of the large-sized fragments in one read and then comprehensively characterize complex genomic loci, such as extended repeats, or resolve sections of unexpected sequence that have been rearranged within the targeted region.

Although the cost of nanopore sequencing is higher than that of NGS, it can produce significantly longer read lengths and walk through traditionally difficult sequence regions which cannot be effectively resolved by NGS. An effective enrichment method suitable for the nanopore sequencing platform would greatly extend the platform’s application range. Johannes et al. (Dapprich et al., 2016) supplied a target region enrichment method that can successfully obtain a large-length template; however, this method is very time-consuming and the yield of products is so low that it cannot be applied to the third-generation sequencing platform. Here, we present a method of target-region enrichment in which the adaptor ligation products are extended with two round nested-primers and then PCR with the newly introduced universal primers. This target-region enrichment method can produce large-sized products. Our experimental data reveal that the average length of data was more than 1 KB, with some read lengths reaching 7–8K. This is of great advantage for the sequencing of complex regions and structural variation searching. As the last step of this method is PCR, it is possible to obtain enough DNA to meet the needs of ONT ligation sequencing.

The spacing parameter in this study was not optimized in primer design but only set at a value of 1 KB, based on subjective judgment, so that the final number of primers would be relatively large. In fact, this parameter may be worth optimizing to help control the synthesis cost of primers and interference between them. Antholigo, which was adopted as the primer design method by Johannes et al. (Dapprich et al., 2016), was actually what we wanted to use at the beginning. However, due to its lowest spacing of 5 KB, we were concerned that the amplification efficiency of such a large segment of DNA would be low, so we did not use this software. However, it might be possible to use the software directly if amplification was performed using polymerases that specialize in long DNA-fragment PCR. Finally, according to the design idea of Antholigo, we designed one primer per 1 KB with Primer3, and then evaluated the dimer between primers. If there was a serious dimer, we eliminated and redesigned it.

The RSA method provided in this paper could not only be applied to the 22q11 region involved in this study but also extended to enrichment in other larger regions. The RSA method can capture long DNA segments from genomic DNA samples, which may include entire adjacent target regions—including exon, intron, and intergenic regions. It is thus a feasible nanopore sequencing method for those complex regions, such as large segment insertions, deletions, ectopics, and other structural variations.

## Conclusion

We have described a feasible nanopore sequencing method for the 22q11.2 deletion, which may be extended to the enrichment in other larger regions. RSA was able to deliver adequate coverage (>98%) and adequate long reads (average length >1 Kb) throughout the 22q11.2 region. The long ONT sequencing reads, derived from three umbilical cord blood samples, have facilitated the breakpoint finding of the 22q11.2 deletion, which was validated by Sanger sequencing.

## Data Availability

The datasets presented in this study can be found in online repositories. The names of the repository/repositories and accession number(s) can be found in the article/Supplementary Material.

## Abbreviations

RSA, region-specific amplification.
