# Supplementary figures and images for: A method of large DNA fragment enrichment for nanopore sequencing in region 22q11.2
Source: Front Genet. 2022 Oct 31;13:959883. doi: 10.3389/fgene.2022.959883 (PMC9659874; doi:10.3389/fgene.2022.959883)

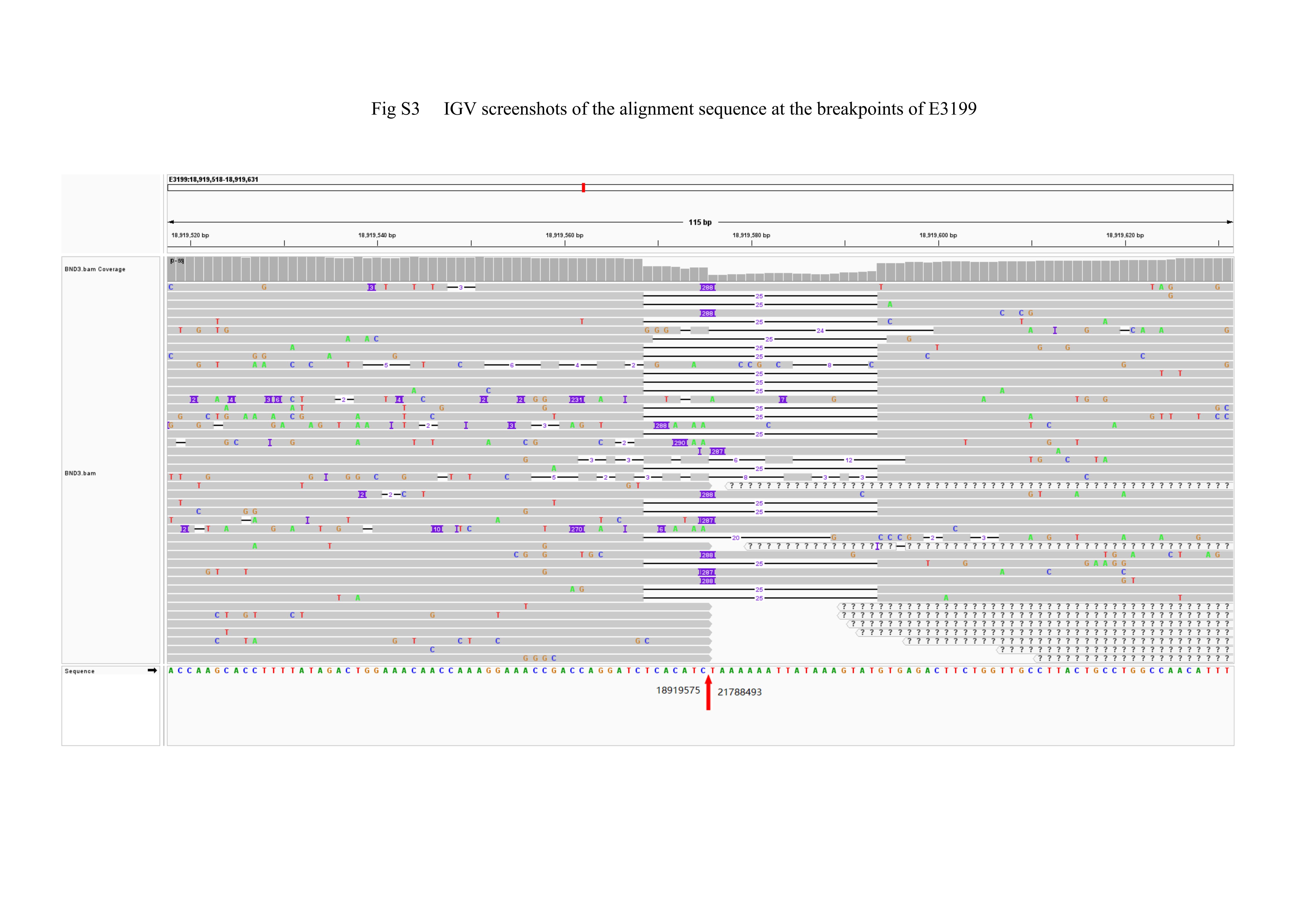

Supplement: Supplementary file 2 [file Image3.JPEG]

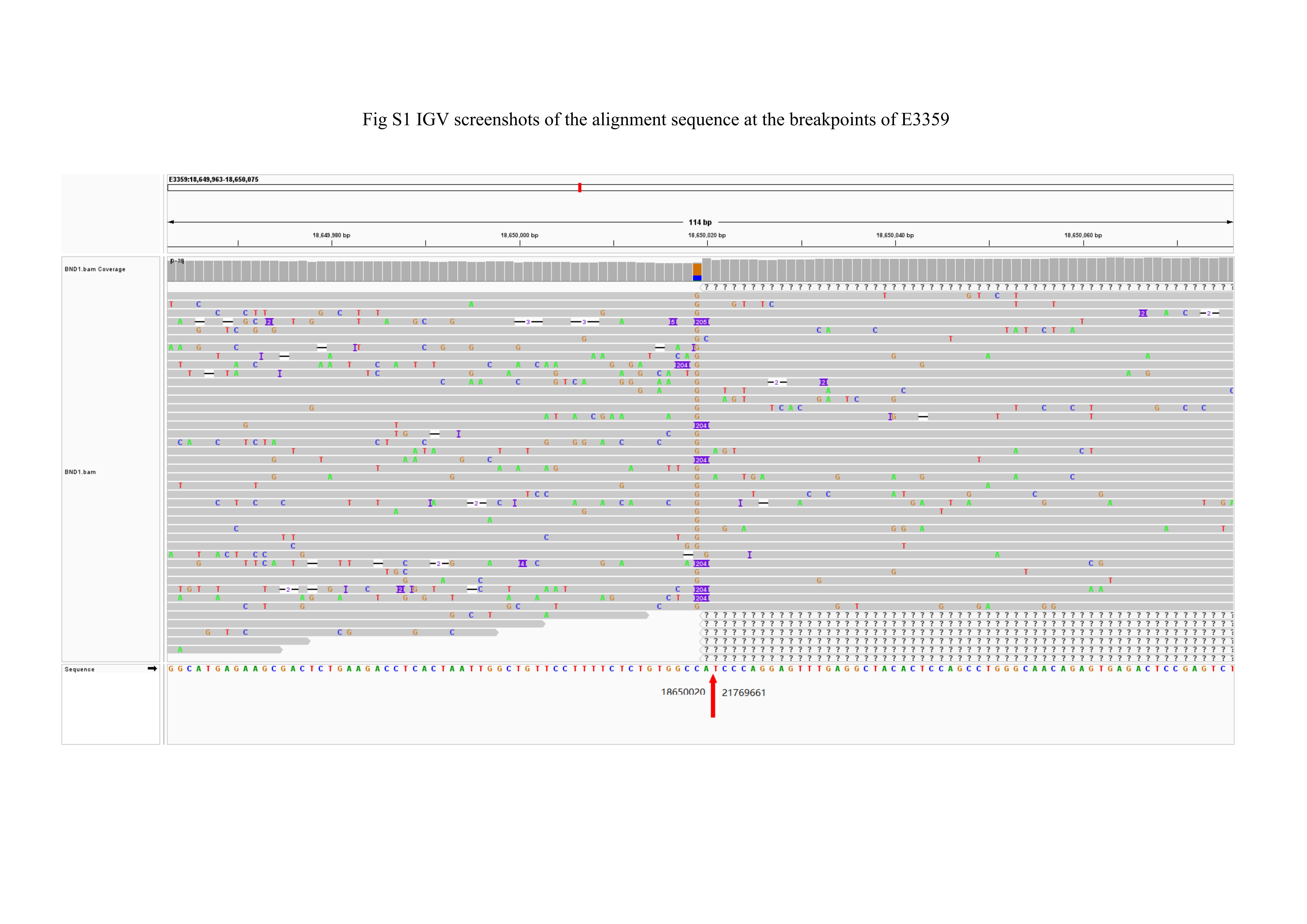

Supplement: Supplementary file 4 [file Image1.JPEG]

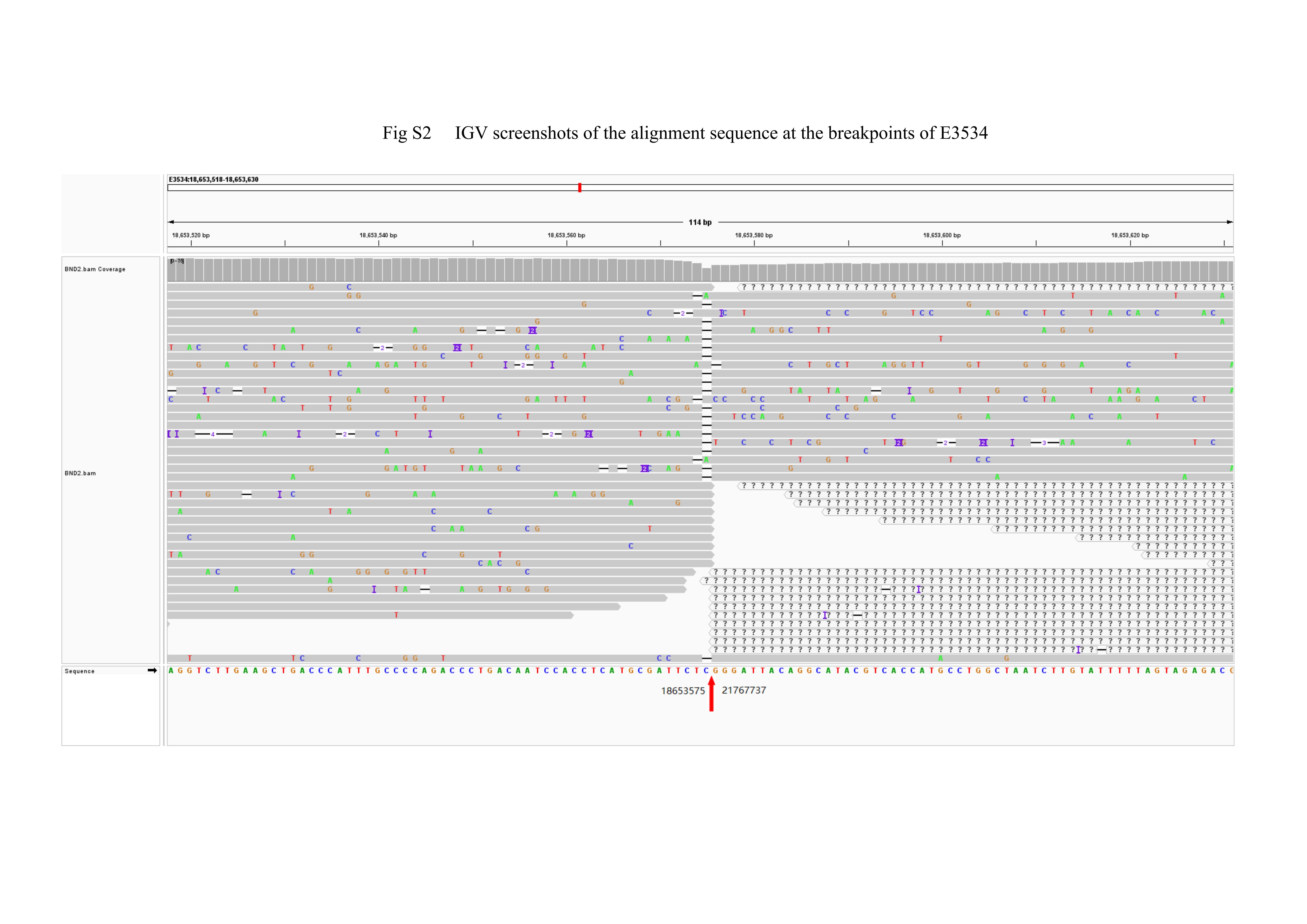

Supplement: Supplementary file 5 [file Image2.JPEG]
